# Commentary: Sex-based medicine in psoriatic arthritis: Lessons learned from machine learning-based prediction models

**DOI:** 10.3389/fimmu.2022.1038270

**Published:** 2022-10-03

**Authors:** Rubén Queiro

**Affiliations:** Rheumatology Division & Instituto de Investigación Sanitaria del Principado de Asturias (ISPA). Hospital Universitario Central de Asturias & Oviedo University School of Medicine, Oviedo, Spain

**Keywords:** psoriatic arthritis, sex, gender, machine learning, outcome

## Introduction

There is a growing interest in sex-based medicine as evidenced by the recent review by Bragazzi et al. referring to the current knowledge of sex-specific differences related to psoriatic arthritis (PsA) (1). Both sex and gender are important drivers of epidemiology, clinical manifestations, prognosis, as well as response to treatment, in diseases suffered by humans (2). These considerations are, of course, applicable to the field of autoimmune diseases, where, for example, we know that most autoimmune connective tissue diseases are more prevalent and severe in women than in men (3). In general, in diseases such as SLE that mostly affect young women of reproductive age, it is easy to infer that biological factors linked to sex are key in the pathogenesis of the disease (3). However, in other diseases such as PsA where there is no clear prevalence of the disease between men and women, the interactions between sex or gender with the different facets of the disease are not so apparent (1, 4).

Traditionally, the majority of studies that have analyzed the differences between men and women with PsA, indicate a greater impact on quality of life, worse functional capacity, worse evolution, and a worse response to treatment, in women compared to men with this entity (1, 4). Likewise, women with PsA tend to be less likely to achieve treatment goals after systemic therapies (1, 4). All this is a clear call to action to go beyond the simple analysis of the differences between men and women with PsA, to delve deeper into the bases that support these differences.

## Sex-based medicine in psoriatic arthritis: what can we learn from machine learning-based prediction models?

Perhaps one of the most useful tools to delve into the bases that support these sex-based differences in PsA lies in modern technologies based on artificial intelligence (AI), and their potential applications as predictive methodologies of the clinical course of PsA or the response to current treatments against this illness (5). If, in addition, we apply these techniques to patients at an early stage of the disease, with rigorous prospective follow-up, we would be taking important steps on the road to elucidating the reasons for differences based on sex or gender in PsA. In that sense, some recent studies are contributing to a better understanding of the evolution of PsA, as well as the role that sex can play in this regard. For example, a recent prospective study conducted in subjects with recent-onset PsA (before the natural history of the disease is modified by drugs), evaluated patient- and disease-related variables linked to the probability of achieving an MDA (minimal disease activity) response both at the first- and two-year follow-up visit. The 4 variables that, in hierarchical order according to the SHAP^1^ 1The SHAP (SHapply Additive exPlanations) method is a technique for explaining the output of artificial intelligence models. It assigns a SHAP value to each value of each variable of each data item in the evaluation set according to the degree to which it affects the prediction of the model (the higher the SHAP absolute value, the more the data item affects the prediction) and to the direction in which it affects the prediction of the model (a positive SHAP value indicates a positive effect on the prediction, that is, it contributes to the prediction having a higher value, and the opposite with a negative SHAP value). (SHapply Additive exPlanations) method, had the greatest predictive weight were pain, disease impact measured by the PsAID (PsA impact of disease), disease activity according to the patient, and physical disability according to the HAQ (health assessment questionnaire), while the predictive weight of sex was quite marginal (6). In another study from the same cohort, the variables predicting severe disease were pain, treatment with conventional synthetic drugs, clinical form at diagnosis, high C-reactive protein, arterial hypertension, and psoriasis affecting the gluteal cleft and/or perianal area. Regardless of sex, the mean values of the measures of validity of the machine learning algorithms were all ≥ 80% (7).

Recent data from the ReFLAP (Remission/Flare in PsA) study show that women with PsA score worse on enthesitis, pain, physical function, fatigue, and disease impact. In addition, women were less likely to achieve treatment targets. The factors associated with high-impact disease (PsAID ≥4) in ReFLAP were female sex, enthesitis, tender joint count, and comorbid conditions (8). The reason for this apparently worse disease outcome in women with PsA is not clear, although the various options include psychosocial factors, hormonal factors, age at onset, and genetic factors (1, 4, 9) However, most of the studies carried out so far have not considered the possibility of confounding factors that could disentangle this association. A novel study of patients with recent-onset PsA has addressed this issue. The dataset was generated using data for each patient at the 3 study visits (baseline, first year, and second year of follow-up) matched with the PsAID values at each of the 3 visits. Once variables associated with both PsAID ≥4 and sex were selected, those that led to a difference of >10% between the adjusted and crude estimations were identified as potential confounders in the association between sex and high-impact disease. Female sex was associated with PsAID ≥4 in the crude analysis (OR 1.82). In line with the criterion of a difference of >10% between the crude and the adjusted OR, the potential confounders in the association between sex and PsAID were HAQ, pain, patient global assessment of disease, level of physical activity, and joint pattern at diagnosis (**Table 1**). After adjustment for these variables, no statistically significant association was observed between female sex and PsAID ≥4 (10).

**Table 1:** Variables associated with high-impact disease in a prospective cohort of recent-onset PsA patients.

Variable	Regression coefficient	95% CI
**HAQ**	10.39	(7.78, 13.01)
**Pain in the previous week**	5.67	(4.02, 7.32)
**Low educational level**	-2.06	(-3.52, -0.61)
**High level of physical activity**	1.22	(0.16, 2.28)

Regardless of gender, these were the main variables associated with a high-impact disease. When the random forest–type and XGBoost machine learning algorithms were trained with these 4 variables, the order of importance (from more to less) attributed by most of the models according to the values of feature importance was as follows: HAQ, pain in the previous week, educational level, and physical activity during the previous week. The mean values of the measures of validity of both types of machine-learning algorithms were all ≥85%.

PsA, psoriatic arthritis; CI, confidence intervals; HAQ, health assessment questionnaire.

## Discussion

There exists methodological heterogeneity and paucity of data concerning sex-specific differences, in terms of the specific population studied, study design, and the PsA diagnostic criteria utilized (1). Although many studies point to more severe and impactful disease in PsA women, when modern AI-based predictive technologies are used in rigorously controlled prospective cohorts, these differences are no longer as apparent (7, 8, 10). Therefore, as Braggazzi et al. stated in their review, “harmonizing and reconciling these discrepancies would be of crucial importance in achieving the ambitious goals of personalized/individualized medicine and further standardized meta-data and Big Data could help disentangle and elucidate the precise mechanisms of underlying potential PsA sex-specific differences”. Thus, more studies are still needed, with a better design and with a large enough number of patients to shed light on this aspect of PsA. Both the review by Bragazzi et al. (1), as well as other recent works (4), indicate an urgent need for more PsA studies in this new field of gender- and sex-based personalized/individualized medicine. Machine learning-based modeling can help with this task.

## Author contributions

The author confirms being the sole contributor of this work and has approved it for publication.

## Conflict of interest

The author declares that the research was conducted in the absence of any commercial or financial relationships that could be construed as a potential conflict of interest.

## Publisher’s note

All claims expressed in this article are solely those of the authors and do not necessarily represent those of their affiliated organizations, or those of the publisher, the editors and the reviewers. Any product that may be evaluated in this article, or claim that may be made by its manufacturer, is not guaranteed or endorsed by the publisher.
